# Validation of Software for Patient-Specific Real-Time Simulation of Hepatic Radiofrequency Ablation

**DOI:** 10.1016/j.acra.2021.12.018

**Published:** 2022-01-14

**Authors:** Eric K. Hoffer, Andrea Borsic, Sohum D. Patel

**Affiliations:** Director of Interventional Radiology, Dartmouth Hitchcock Medical Center, Boston, Massachusetts; One Medical Center Dr., Lebanon, New Hampshire 03755. CEO NE Scientific LLC, Boston, Massachusetts; Geisel School of Medicine at Dartmouth, Hanover, NH 03755

**Keywords:** liver neoplasms, Software, Ablation techniques, Computer-assisted therapies

## Abstract

**Rationale and Objectives:**

CT-guided radiofrequency ablation (RFA) is a potentially curative minimally invasive treatment for liver cancer. Local tumor recurrence limits the success of RFA for large or irregular tumors as it is difficult to visualize the tissue destroyed. This study was designed to validate a real-time software-simulated ablation volume for intraprocedural guidance.

**Materials and Methods:**

Software that simulated RFA physics calculated ablation volumes in 17 agar-albumin phantoms (7 with a simulated vessel) and in six in-vivo (porcine) ablations. The software-modeled volumes were compared with the actual ablations (physical lesion in agar, contrast CT in the porcine model) and to the volume predicted by the manufacturer’s charts. Error was defined as the distance from evenly distributed points on the segmented true ablation volume surfaces to the closest points on the corresponding computer-generated model, and for the porcine model, to the manufacturer-specified ablation volume.

**Results:**

The average maximum error of the simulation was 2.8 mm (range to 4.9 mm) in the phantoms. The heat-sink effect from the simulated vessel was well-modeled by the simulation. In the porcine model, the average maximum error of the simulation was 5.2 mm (range to 8.1 mm) vs 7.8 mm (range to 10.0mm) for the manufacturer’s model (*p* = 0.009).

**Conclusion:**

A real-time computer-generated RFA model incorporated tine position, energy deposited, and large vessel proximity to predict the ablation volume in agar phantoms with less than 3mm maximum error. Although the in-vivo model had slightly higher maximum error, the software better predicted the achieved ablation volume compared to the manufacturer’s ablation maps.

## INTRODUCTION

This study was performed to validate a novel software to intraoperatively guide radiofrequency ablation (RFA) of liver neoplasms. Hepatocellular carcinoma (HCC) is the fifth most common cancer and the third leading cause of cancer deaths globally, with a rising incidence in the US (1,2). The most minimally invasive potentially curative therapy is percutaneous tumor ablation. For lesions less than 3 cm diameter, ablation can provide up to 70% 5 -year survival, which is similar to surgical resection, and superior to the palliative regional or systemic therapies (1,3).

Radiofrequency ablation, one of the earliest, most commonly used, and most studied methods, involves image-guided direct application of RF energy to a target volume of tissue that includes the tumor and a 5 mm margin, with the goal of leaving no viable tumor cells (1–4). However, in practice, imprecise alignment of the ablation zone and the target results in incomplete destruction of the target that results in local recurrence rates of 26.4% for HCC tumors 3–5 cm in diameter (5). This error rate is largely due to the inability to accurately predict and visualize the achieved ablation zone. The intraprocedural standard to predict the extent of the tissue damage from an ablation is to mentally superimpose the device manufacturers’ idealized map of the area of expected tissue damage onto the 2D cross-sectional images. This is improved by the recent introduction of software that can superimpose the idealized ablation map on multi-planar images (6,7).

Though useful in planning, this approach is limited for confirmation of adequate target coverage in that the manufacturer’s ablation map is an idealized estimate (often from ex vivo non-human models), which cannot account for patient specific variation, such as proximity to larger blood vessels. In an analysis of treatments that resulted in < 3 mm ablation margins, the variation from the expected ablation volume was attributable to vessel proximity in 47% of cases (5).

Immediate identification of an ablation volume, to determine adequacy of a treatment, may be obtained by a contrast-enhanced CT where the ablated tissue does not enhance (8–10). Yet for larger or irregular lesions, if overlapping ablations are necessary, concern for renal toxicity often limits the amount of iodinated contrast-that can be administered safely (6,7).

A method for patient-specific pre-procedure planning, and ablation confirmation that does not require intravenous contrast, is a computer-simulated ablation volume. When calculated from patient-specific data that reflects the energy delivered, such a simulation can account for large vessels and tissue perfusion, and accurately depict the volume of ablated tissue. Until recently, such a simulation took over 20 minutes to calculate, and so had minimal intraoperative utility. Developments in Graphics Processing Unit (GPU) technology now allow this simulation to be computed in real-time (8–10).

The purpose of this research was to validate novel software that calculates a simulated ablation zone in real-time from patient- and procedure-specific data. We hypothesize that this computed model will provide a more accurate estimate of the ablation tissue volume than the manufacturer’s charts.

## MATERIALS AND METHODS

A software library for simulating the RF ablation physics (NE Scientific, Boston, MA, USA) was employed to calculate a simulated ablation volume based on patient-specific factors in bench-top and in-vivo experiments with the purpose of validating the simulation. Specifically, a series of ablations in agar-albumin phantoms, and in in-vivo (porcine) models were conducted. The accuracy of each software-modeled ablation volume was compared with the actual ablation volume (from the physical lesion in agar,and from contrast CT in the porcine model) and with the ablation volume predicted by the manufacturer’s chart.

### Hardware

Ablation was performed with the Boston Scientific LeVeen electrode and RF 3500 power generator (RF 3000, Boston Scientific,Inc., Marlborough, MA, USA). The reference manufacturer’s expected ablation zone was that provided at the time (2005 version). This was the system used as it continues to be our primary RF system based on our 17-year experience. Historically, we found the tines provided relatively accurate estimation of the extent of the ablation perpendicular to the device; further, they anchor the system in place, which is a concern when an oblique access is used, and respiratory motion can displace a straight needle electrode. Due to the potential tine displacement when deployed from their unconstrained symmetric relation to each other, the software was designed to identify and account for the effect of any distortion on the resultant ablation volume.

### Software

The RFA Physics Simulation Library (NE Scientific, LLC, Boston, MA, USA) is a combination of models that account for electrical, thermal, and tissue damage (detailed in Appendix A). The electrical model translates the electrical energy applied to the RFA electrode into the electrical power density dissipated in the tissues. Based on the power density at each point, the thermal model simulates the temporal evolution of tissue temperature. The thermal model can account for the heat-sink effect of blood vessels, perfusion, and the evaporation occurring in the tissues. Subsequently, an Arrhenius model (11) is used to determine tissue damage based on temperature and the time of exposure. The simulation library uses the Finite Element Method (FEM) to discretize the model equations in the spatial domain, and Finite Difference Method (FDM) to discretize the model in the temporal domain.

The current GPU-accelerated simulation of a 5-minute ablation is performed in less than 2.5 seconds using a three-dimensional Finite Element Mesh with more than 1.9 million tetrahedral elements, when run on an NVIDIA Ampere A100 PCIe GPU (NVIDIA, Santa Clara, CA, USA)(9)

### In Vitro Study

#### Phantoms

**Figure 1.**
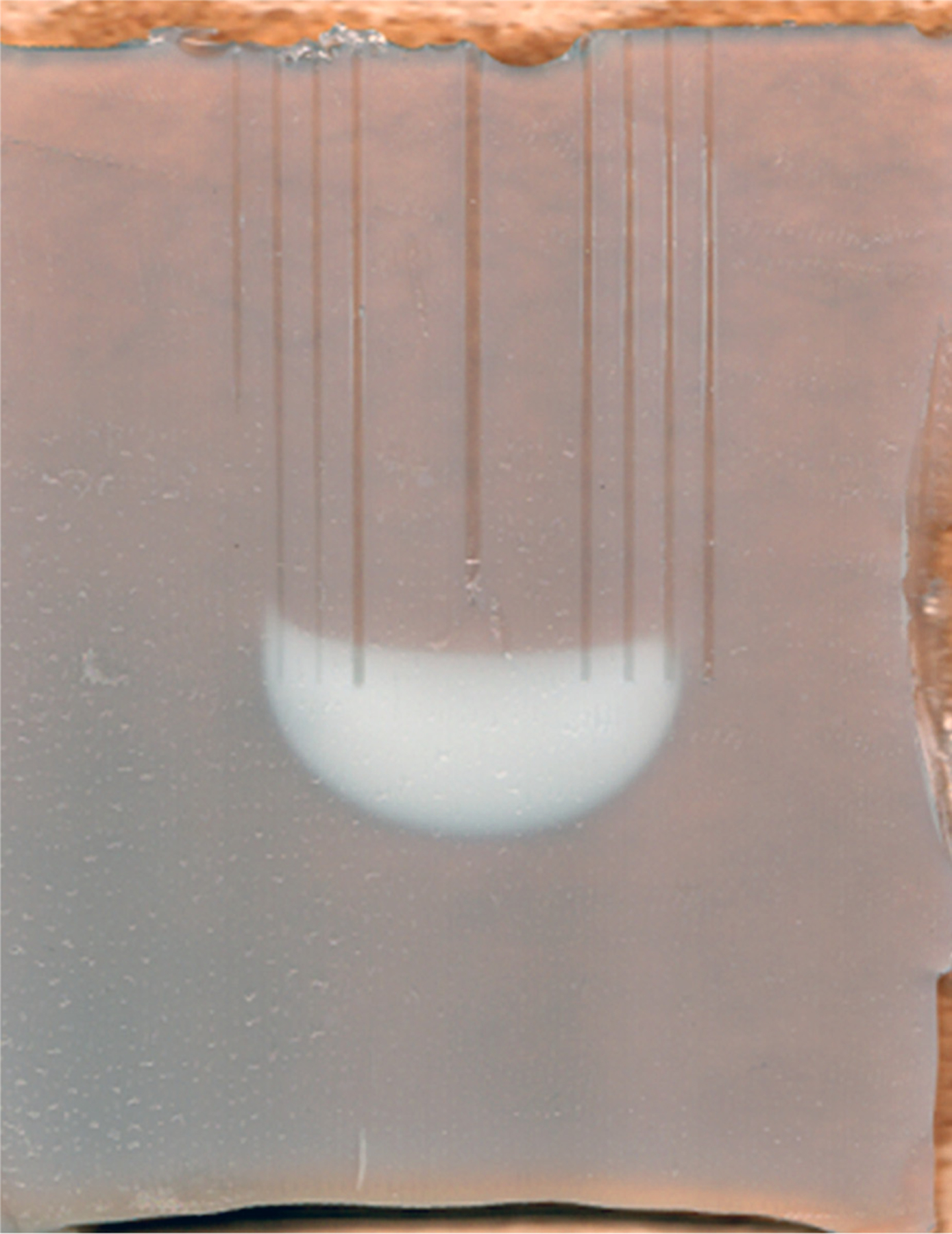
Ex-vivo model: albumin denaturation after radiofrequency ablation: A slice of an agar-albumin phantom (on a brown towel to improve contrast). The vertical track in the center of the slice was left by the shaft of the electrode, the other vertical tracks were left by temperature sensors. The area of denaturation is indicated by the opaque white, distinct from the otherwise semi-transparent color.

The albumin-agar phantom utilized was previously validated with respect to the electrical -thermal heating and thermal porcine liver tissue injury (12,13). When heat denatures the albumin in the phantom there is a thermochromatic change from transparent to opaque-white (Fig 1). This process models tissue necrosis due to heat exposure, a process also based on denaturation of tissue proteins (12).

A 3D printed support was applied to the top of a two-liter glass beaker to hold a LeVeen electrode (Boston Scientific, Inc., Natick, MA, USA) in a fixed position within the agar phantom. A Universal Electrosurgical Pad 9100 Series (3M, Maplewood, MN, USA) was applied to the bottom of the beaker to serve as a return path for the ablation current. The agar-albumin mixture was prepared by mixing 40g of agar (Reagent Grade Agar Powder,CAS# 9002–18-0, Carolina Biological Supply Co., Burlington, NC, USA) in 1.9 liters of cold tap water. The mixture was brought to boil using a stirring hot-plate. Once cooled to 55C, 45g of egg albumin (Organic Valley Egg Whites, La Farge, WI, USA) at 5C were quickly mixed into the agar. Mixing low temperature albumin with agar at 55C produced a mixture which could be poured into the beaker without denaturation of the albumin on contact with the agar.

In addition, phantoms that included a simulated vessel at distances of 25mm, 30mm, and 35mm from the shaft of the electrode were prepared to assess the simulation library’s ability to describe the heat-sink effect. In these phantoms a 5mm diameter channel was created by inserting a plastic straw in the beaker before pouring the agar and removing the straw after the agar was solidified. Water flow at 10.5 cm/s was established with a Watson Marlow 323 (Watson Marlow, Wilmington, MA, USA) pump–similar to the flow measured by the authors with ultrasound doppler in vessels of similar diameter in a porcine model.

After RFA was performed a 3D-printed template allowed repeatable cuts through the agar, and these sections were then scanned on a flat-bed scanner and converted to gray scale such that automatic image segmentation algorithms could be used to segment the boundary of the ablation from the scanned images. Together, these constituted the true ablation volume of the phantom.

#### Simulation

**Figure 2.**
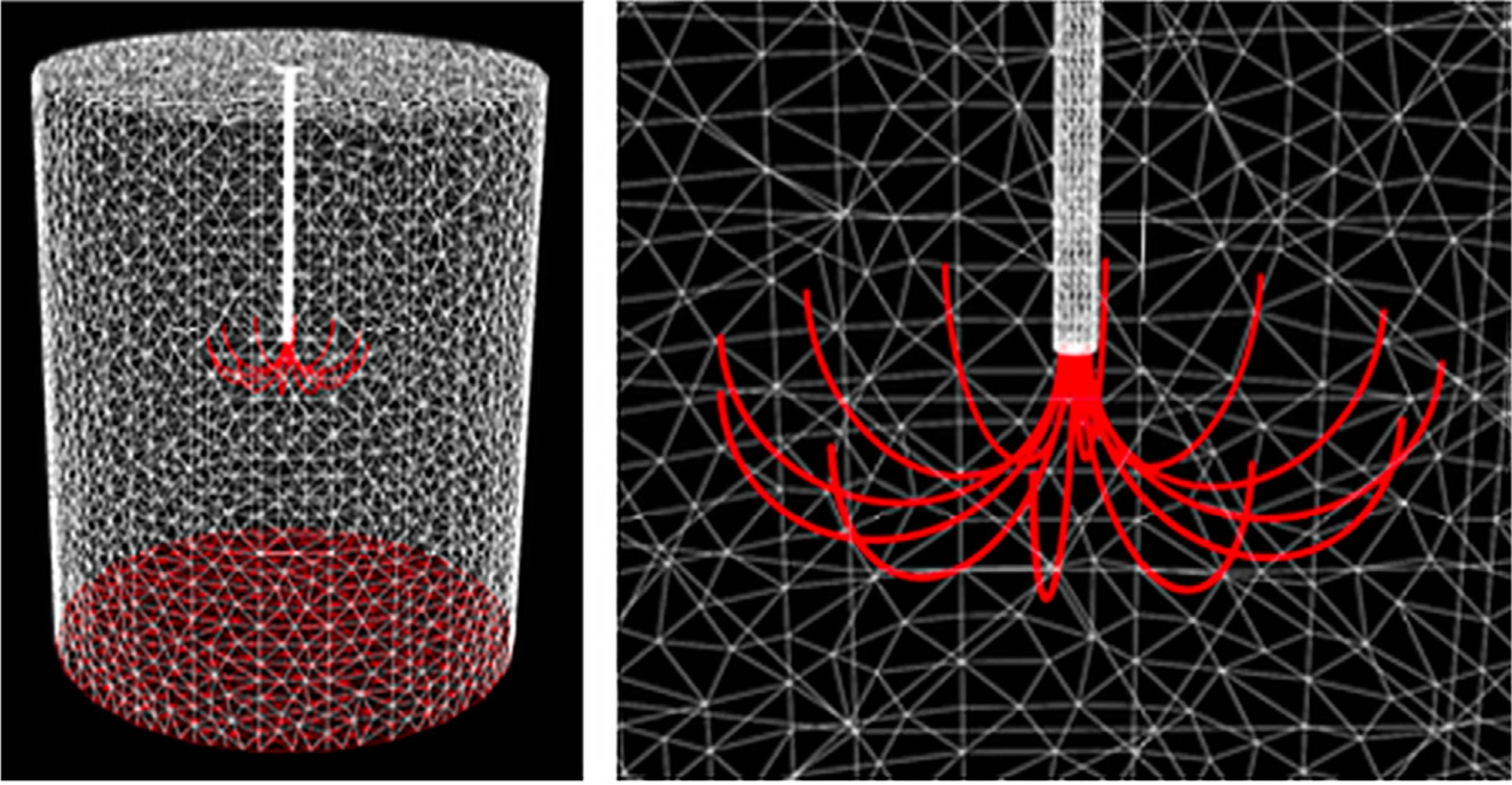
Finite Element Model (FEM) mesh of the phantom model for a 4 cm LeVeen electrode. The red surfaces represent electrodes, which are the tines of the LeVeen electrode, and the circular return pad at the bottom of the phantom. (a) whole model, (b) enlarged at tines.

Investigator-developed routines were used to generate a Finite Element Model of the phantom (Fig 2), including the geometry of the 2 – 4 cm diameter LeVeen electrodes. The high level meshing routines relied on the tetrahedral mesh generator (TetGen, http://wias-berlin.de/software/tetgen/) to discretize the geometry of the phantom, produce meshes of the phantom and embedded electrode, and of the simulated vessel, where present. The FEM mesh of a 4 cm electrode had 65K nodes and 385K tetrahedral elements (detail in Appendix B).

During each ablation a laptop PC was connected to the RF power generator (RF 3000, Boston Scientific,Inc., Marlborough, MA, USA) via a serial cable, and the applied RF power was sampled once per second, and saved to a file using custom developed software (NE Scientific, LLC). The recorded power data was then fed into the RFA physics library, and the ablation volume simulated.

#### Analysis

**Figure 3.**
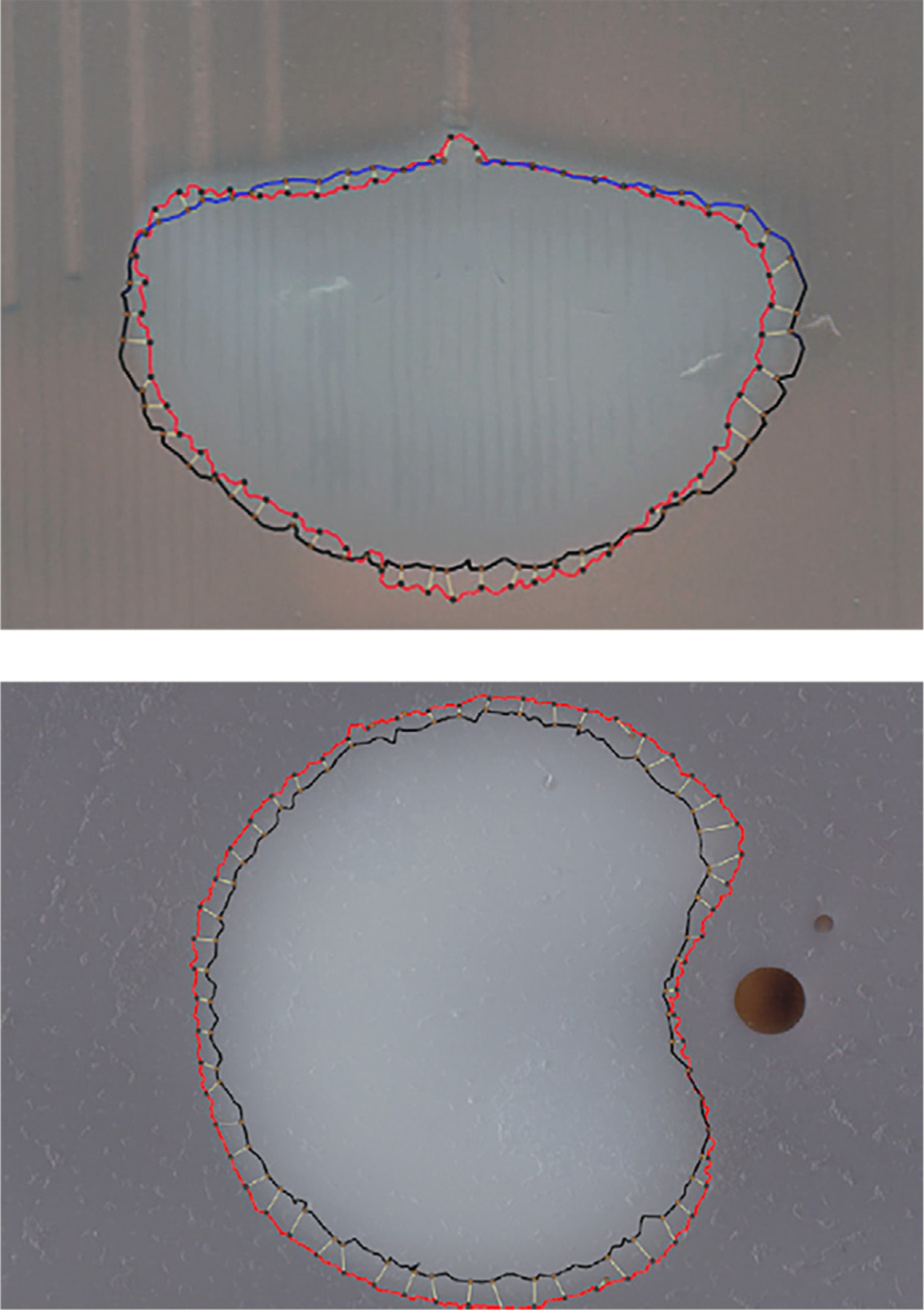
Quantification of the error in the prediction of albumin denaturation volume. The red lines demonstrate the true ablation boundary; dark lines show the computer prediction. (a) a scanned agar longitudinal slice without a simulated vessel. (b) scanned agar axial (perpendicular to long axis of probe) slice with a simulated vessel. The alteration of the ablation contour caused by the presence of the vessel-simulating water channel is evident.

Phantoms without a water channel were sectioned in the coronal plane (Fig 3a) as this plane captured the shape of the ablated volume. Phantoms with a water channel were sliced in an axial plane, as that projection best depicted the heat-sink effect (Fig 3b).

Each section through the agar was converted to gray scale, and a Level Set algorithm segmented the boundary that defined the true ablation profile (14). This was defined by 64 equidistant points, and the distance from each point to the closest point on the registered software-simulated profile were computed (Fig 3). Error was defined as the linear distance between a point on the computer-generated model and the margin of the true ablated volume surface. This data was used to identify the mean and maximum error of the simulated ablation zone.

### In-Vivo Study

After Institutional Animal Care and Use Committee approval, 10 ablations were created in two 45 kg female swine in an operating suite with an incorporated CT (Siemens, Munich, Germany). Pre-procedural contrast-enhanced CT (50 cc/injection, Omnipaque 350 mg/ml, GE Healthcare, Waukesha, WI, USA) was used to visualize the hepatic vasculature and plan multiple ablation sites in the liver at varying proximity to 3 to 10 mm diameter vessels. A 3 cm LeVeen RFA electrode was positioned under CT guidance to obtain non-overlapping ablations. After CT documentation of probe position, the RF power was applied per protocol (Boston Scientific RF 3000 generator) and the power and electrical impedance sampled at 1 second intervals using a laptop PC connected to the RF generator via serial port; data was saved to disk and later fed to the RFA Physics Library to simulate the ablation.

**Figure 4.**
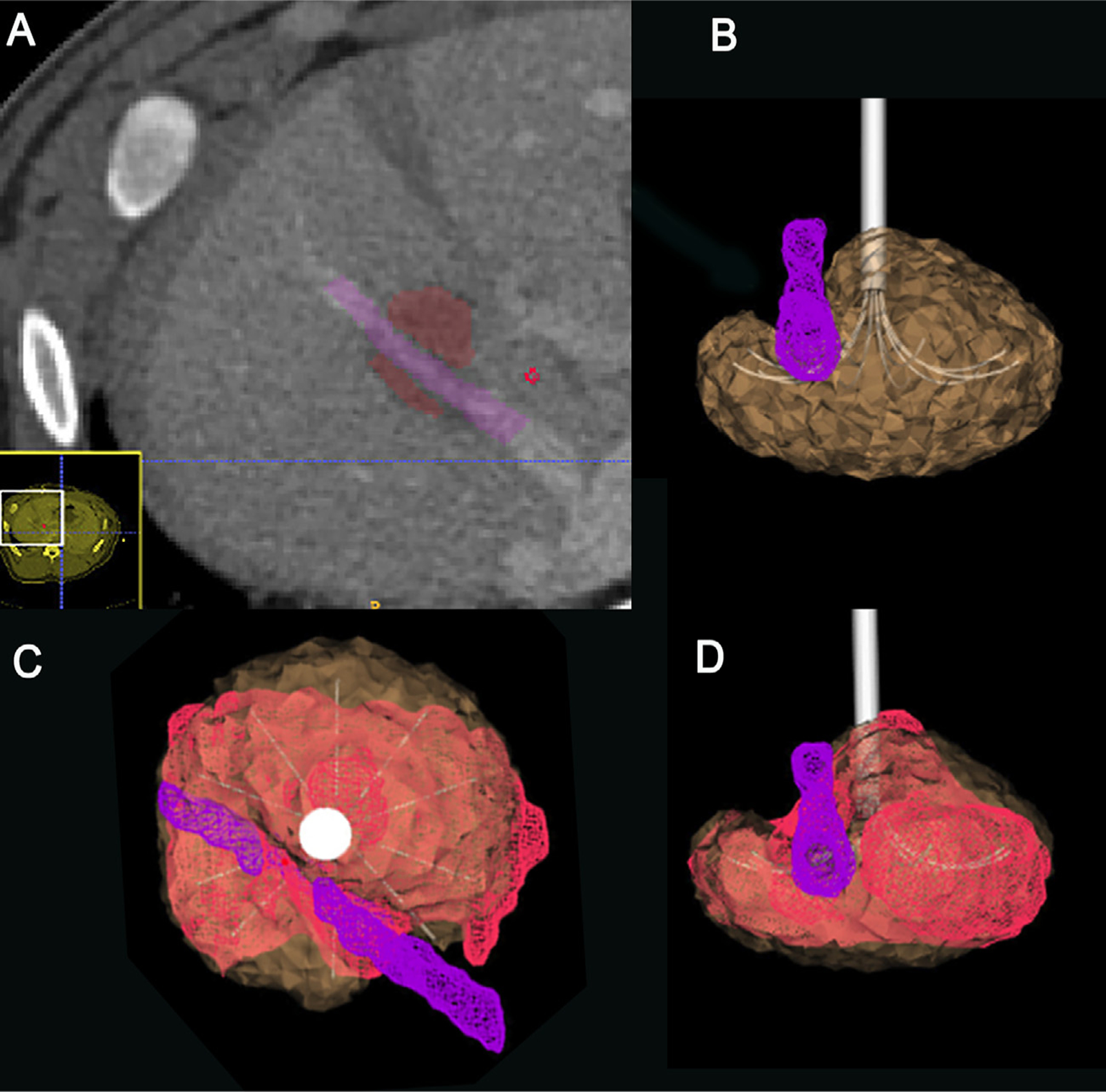
Axial CT after ablation #2 in porcine model and images of segmented volumes demonstrate steps of analysis. (a). the post-ablation contrast CT depicts the true (non-enhanced) ablation volume, which is segmented (red), as is the adjacent hepatic vein (purple). (b). The software generates a simulated ablation volume (brown) given the power applied over time, the device position, and the location of the blood vessel. (c, d). Along the probe and sagittal view of the registered volume models from which the distance difference between the outer surfaces were calculated.

At each site, a post-ablation contrast-enhanced CT scan was acquired, from which the true ablation volume was segmented (Fig 4a). The lesion geometry was segmented manually using ITK-SNAP (http://www.itksnap.org/).

#### Simulations

The software computed lesion geometry was specific to each deployment. Investigator-developed algorithms identified the deployed electrode tine positions in the CT images (as tines can deflect up to 5mm from their ideal position) and generated a Finite Element model of the electrode as deployed. Vessels greater than 3mm diameter within 1cm of the expected ablation were segmented with ITK-SNAP. The electrode and vessel geometry were used to produce 3D tetrahedral finite element meshes with approximately 1.6 million discrete elements that were used by the RFA Physics Library to compute the simulated ablation. The computed ablation volume was registered into the subject coordinates based on the electrode position from the images (Fig 4b).

For comparison purposes, the manufacturer’s probe-specific chart for the expected ablation volume was used to build a 3D computer surface, ie, for a diameter of 3cm, lesion depth proximal along the shaft was 9mm, and distal was 16mm, and a 3D surface was created from the combination of the proximal and distal half-ellipsoids.

#### Analysis

For each ablation, error metrics were computed using the distance between approximately 10,000 evenly distributed points on the segmented ablation volume surfaces to the closest points on the corresponding computer-generated model (Fig 4c–d), and separately to the 3D surface generated from the manufacturer’s chart. Significance was assessed by two-sided paired sample t-test, at *p* <0.05.

## RESULTS

### In-Vitro Study

Ten agar-albumin experiments were conducted without a simulated vessel, and seven were conducted with a simulated vessel with varying offset from the center of the phantom to simulate the heat-sink effect. The summary results of each experiment without and with simulated vessels are tabulated in Table 1 (detail in Appendix C, Table C1 and C2). The maximum error for a software simulated ablation volume ranged from 1.6 to 4.9 mm in the phantoms. The defect in the ablation volume due to the flow channel was well modeled by the simulation (Fig 3b).

### In-Vivo Study

Ablated contours from six of the ten ablations could be manually segmented. The other four were not analyzed due to unreliable identification of margins due to local hemorrhage distortion or inadequate contrast. The post-ablation contrast enhanced CT segmented ablation volume surface was compared with both the manufacturer’s predicted ablation volume and the software-calculated ablation volume. Mean and maximum errors were improved with the computer model vs manufacturer’s chart for all lesions. In the porcine model, the maximum error for a software-simulated ablation volume ranged from 3.6 to 8.1 mm, which were less than the 6.3 to 10.0 range for the manufacturer’s model (*p* = 0.009) (Table 2, Detail in Appendix C Table C3).

## DISCUSSION

**Figure 5.**
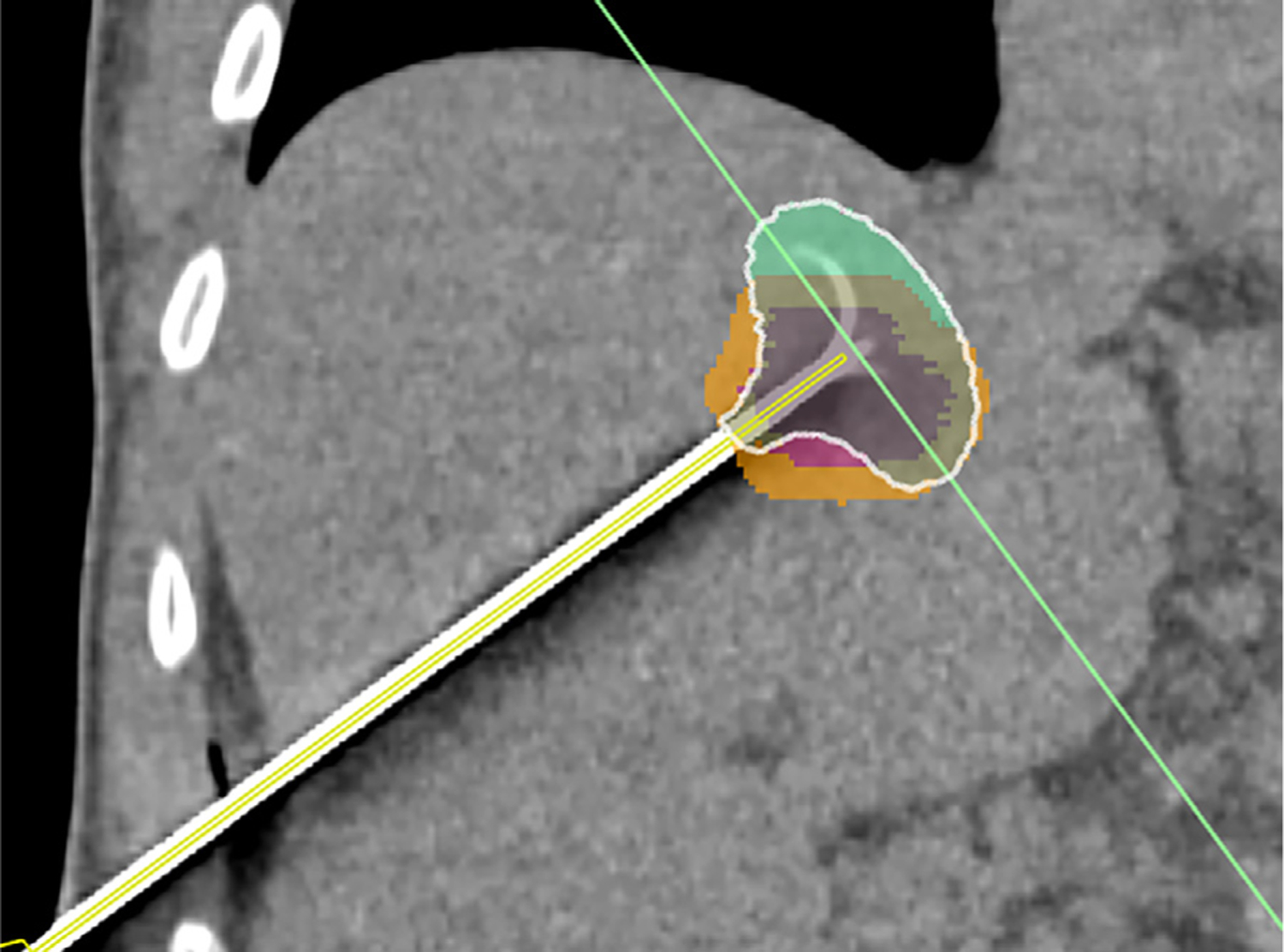
Clinical application of the software. Coronal image depicts white RFA probe in liver, with segmented tumor (red), calculated 5 mm margin (yellow), and ablation volume calculated from energy administered (green). Treated portion of target tissue is darkened.

An ablation zone model produced by computation of patient-specific procedural and anatomic parameters demonstrated improved accuracy compared to manufacturer’s model. The contour of the computed ablation geometry closely followed that of the “true” burn profile, as evidenced by visual analysis (Fig 3) as well as quantitative matching errors (Tables 3,5). The accuracy of this volume estimate is important, as it is the interventionalist’s primary indication as to whether an ablation achieved the target coverage required to minimize the risk of local recurrence (tumor and 5 mm margin) (Fig 5).

Prior work demonstrated the efficacy of a GPU-accelerated finite element simulation of Penne’s bioheat model to predict the lesion created by RFA (15). That study used retrospective data to simulate a clinical environment, and found a reasonable accuracy based on mean relative volume deviation of 14% and surface error of 2.4 mm. The current model had an average surface error of 1.1 mm. The current analysis was more conservative regarding the assessment of accuracy: Average errors may underestimate the risk of tumor recurrence, as focal areas where the ablation yields a less than a 5 mm margin may be missed, yet entail an increased risk of recurrence (16,9,17). Evaluation of the maximum error (at any point on the surface) of the model would better reveal that risk.

Though improved over the manufacturer’s charts, the software produced in vivo maximum errors up to 8 mm. As in any clinical setting, where motion cannot be eliminated, some error may be due to segmentation and registration error, as well as heat transport by vapor. When a vapor heat transport model was incorporated, the maximum discrepancy was reduced to 4.7 mm.

A primary limitation to the albumin-agar phantom was the lack of a perfusion component, and so that aspect of the simulation could not be validated. However, the electrical and thermal properties of the phantom were similar to liver (Appendix B, (18)), so the calculations with regard to the electrical power density distribution and the temporal evolution of temperatures driven by the dissipated power density could be validated in the agar model. Tissue perfusion was a potential source of error in the swine model, as it was estimated without variation within different portions of the swine liver. In that the error between simulated and actual ablation volumes was not correlated with the location of large vessels, we infer that the increased error in the swine model was due to lack of an accurate perfusion measurement.

This raises the potential limited applicability to other clinical scenarios in the cirrhotic patient population; for example, obese patients with steatohepatits may have different liver tissue heating properties. Lesions adjacent to occluded or slow-flowing large veins should have less ‘heat sink’ effect, and larger ablation volumes. Further, in the clinical setting, patient motion or iatrogenic deformation of the liver (eg. with the use of artificial ascites) may limit the effectiveness of the automatic rigid registration, and therefore require time-consuming manual registration for the image alignment to accurately project the simulation on the CT image. These patient-specific variables that are potentially unaccounted for are currently under investigation in a clinical validation study.

The studied software simulation model is limited to RF ablation, in that the equations describe the heat created from the applied RF energy. However, given a modality-specific mathematical model (ie, microwave, cryoablation, or irreversible electroporation) the software would be able to rapidly calculate a simulated treatment volume. Equations that model microwave-mediated ablation are being validated. The remainder of the software that supports a user -friendly graphic display of the 3D multiplanar imaging, target segmentation, and virtual probe placement to plan trajectory and visualize the expected ablation volume are readily applicable to other systems.

Ablation of the entire target (tumor and margin) is correlated with a reduction in local recurrence rates (1–3,9). Methods reported to reduce recurrences include embolization prior to ablation, the simultaneous use of multiple applicators, or early evaluation and re-treatment for residual viable tumor (19,20). Less costly, more efficient approaches focus on improvements in the accuracy of the ablation procedure. These include 3D stereotactic targeting to get the ablation probe to a precise target site, confirmation of the electrode targeting through fusion of the tumor images with ablation probe images, projection of an accurate ablation volume (for planning), or fusion with a contrast-enhanced segmented actual ablation zone (for assessment of the adequacy of the treatment) (17).

This prospective study focused on validation of the software simulation of an RFA ablation volume in the liver—as a more accurate replacement for the manufacturer’s charts– that does not require an additional IV contrast administration. This simulation is at the core of a CT-ablation guidance package that projects the segmented tumor and desired margin on 3D multiplanar images throughout the procedure. A virtual probe and expected ablation volume can be placed and visualized for planning. The virtual probe acts as an ideal path with which electrode advancement can be assessed. Once in place, after image registration, the target is displayed simultaneously with the expected ablation volume for that electrode position. And then after the ablation, the energy administered is used to calculate the simulated ablation volume, which is displayed such that any portion of the target (tumor and margin) not encompassed by the ablation volume was clearly evident (Fig 5).

In conclusion, this study evaluated a real-time computer-generated ablation model that incorporated tine position, actual energy deposited, and large vessel proximity. This simulation predicted the ablation volume in agar phantoms with less than 3mm maximum error; and despite slightly higher maximum error, the software better predicted the achieved ablation volume in the in vivo models as compared to the manufacturer’s ablation maps. We hypothesize that intraprocedural visualization of this computed simulated ablation volume from imparted RF energy will improve the accuracy and so, efficacy of RFA. Incorporation of such visualization software may enable improved accuracy for the multiple ablations required for larger lesions without added contrast enhanced imaging. A clinical trial in progress will further evaluate this experimentally validated ablation model in a full-function software package that incorporates the required segmentation, visualization, and registration for real-time guidance of the ablation procedure.

## Figures and Tables

**TABLE 1.** Summary of In-Vitro Agar Phantom Results

	n	Avg Mean Error	Range	Avg Max Error	Range
Agar phantom	10	0.92	0.6−1.8	2.5	1.6−4.9
Agar + vessel	7	0.92	0.8−1.7	2.8	1.8−4.0

The error is the distance between the computed ablation surface and the actual (segmented agar slice) ablation surface for each ablation. The mean error is that error averaged for all the point comparisons that comprise each ablation volume. The average mean error is the average of those mean errors across all the ablation volumes (see Appendix C, Table C1, Table C2 for detail).

**TABLE 2.** Summary of In-Vivo Porcine Model Results

Model	*n*	Error (mm)			
		Average	Range	Average	Max Range
Computed	6	1.1	0.9 − 1.5	5.2	3.6 − 8.1
Manufacturer’s Chart	6	2.5	1.9 − 3.1	7.8	6.3 − 10.0
*p*-value		0.003		0.009	

All ablations were performed with a 3 cm probe. Average errors refer to the distance between the computed or manufacturer’s predicted ablation volume surface and the actual ablation volume surface as obtained from segmentation of the contrast-enhanced defined CT ablation volumes. Average max is the average of the maximum error across the 6 ablations (see Appendix C, Table C3 for detail).

## APPENDICIES

### Appendix: A. The Calculation Model

The electrical model translates the voltage applied to the RFA electrode into the electrical power density dissipated in the tissues. Based on the power density at each point, the thermal model computes the temporal evolution of tissue temperature. The thermal model can include the heat-sink effect of blood vessels, the heat-transport represented by perfusion, and local heat transport from water evaporation and subsequent migration of the vapor.

The simulation is driven by the Penne’s bioheat equation (21), which describes the evolution of the temperatures to which the tissues are exposed during RF energy deposition:

(A1)
$$\rho C\frac{\partial T}{\partial t}=\nabla \cdot k\nabla T+Q_{RF}+Q_{B}$$

where *ρ* is the density of the tissue, *C* is the specific heat capacity, *T* is the temperature, *t* is time, *k* is the thermal conductivity of tissues, *Q*_*RF*_ is the electrical power density deposited by the application of RF energy, *Q*_*B*_ is a term that models any biological source or sink of heat (like the heat sink effect of vessels and perfusion). Equation (A1) allows computation of the evolution of temperatures at the ablation site when the input parameters are specified. The parameters *ρ*, *C* are functions of the tissues, as is *Q*_*B*_*, Q*_*RF*_ depends instead on the applied RF energy, and can be calculated using the Laplace equation:

(A2)
∇⋅σ∇u=0

where σ is the electrical conductivity of tissues and *u* is the electrical potential that develops in the tissues under the effect of the ablation probes. The multiple ablation probes are described with the boundary condition:

(A3)
$$V_{l}=u+zc_{l}\sigma \frac{\partial u}{\partial n}$$

where *V*_*l*_ is the voltage applied to the *l − th* probe, with *l* = 1…*L* where *L* is the number of probes, *zc*_*l*_ is the contact impedance of the *l − th* probe, and $\hat{n}$ is the normal to the surface of the probe. Equations (A1), (A2), and (A3) are discretized spatially with the Finite Element Method (22) and temporally with the Finite ifference Method (23) resulting in the estimation of the temperatures T at the ablation site and over the duration of the ablation.

The computed temperatures are fed into a tissue damage model to determine which tissues will be necrotized, and hence the ablation volume.

The tissue damage is modeled with the Arrhenius equation (11):

(A4)
$$\Omega =\int_{t_{S}}^{t_{F}}Ae^{\frac{-E_{a}}{RT\left(t\right)}}dt$$

where Ω is the tissue damage, a function of space which takes a value of 0 where no tissues are damaged, and of 1 where tissue death is certain; *A* is the Arrhenius pre-exponential factor for the tissues, *E*_*a*_ is the Arrhenius activation energy for the tissue, *R* is the gas constant, *T* are the temperatures computed from (A1), and *t*_*S*_ and *t*_*F*_ are respectively the times at which the ablation is started and finished.

The scalar field Ω that results from (A4) takes therefore values in the range [0,1]. It is common to consider the tissues with an Ω value > 0.9 to be necrotized. The iso surface Ω = 0.9 describes the computed ablation volume resulting from the activation of the *L* electrodes in the system.

The simulation library additionally implements a proprietary model which accounts for the evaporation effects occurring during the ablation. The heat sink effect of vessels is simulated with a convective boundary condition at the interface between the phantom or the tissues and the vessel.

### Appendix B. Model Parameters

The FEM mesh illustrated in Fig 3 is a model of a 4cm electrode, which has 65K nodes and 385K tetrahedral elements. In Fige 3 the electrically active surfaces, the electrode tines and the return pad, are represented in red. The density of the mesh was controlled to be coarser on the boundary, and much finer near tines, where spatial variations in electric field and temperature are larger, and a finer discretization is needed. In terms of boundary conditions, an adiabatic condition was applied to external surfaces of the mesh, resulting in no exchange of heat with the environment, as verified to be the case in practice, given the use of a thermally insulating box to house the phantom.

The following parameters were used to describe the properties of the phantom:

Electrical conductivity: approximately 0.21 S/m - measured for each phantom Contact impedance of tines: 0.001 Ohm m^2 - fitted to data Thermal conductivity: 0.55 W / (m K) Specific heat capacity: 4181 J (Kg K) Density: 999.97 kg/m^3 Arrhenius A constant: 6.17e71 Arrhenius Ea constant: 4.80e5 Convection coefficient at the interface phantom / vessel = 5000 W /(m^2 ⋆ K) Prefusion rate = 0 mL/(min · 100 mL)

The following parameters were used to describe the properties of porcine liver tissues:

Electrical conductivity: 0.12 S/m Contact impedance of tines: 0.001 Ohm m^2 - fitted to data Thermal conductivity: 0.512 W / (m K) Specific heat capacity: 3816 J (Kg K) Density: 999.97 kg/m^3 Arrhenius A constant: 9.4e104 Arrhenius Ea constant: 6.7e5 Convection coefficient at the interface phantom / vessel = 5000 W /(m^2 ⋆ K) Prefusion rate = 108 mL/(min · 100 mL)

### Appendix C. Study Data

Maximum errors refer to the distance between the computed or manufacturer’s ablation volume surface and the actual ablation volume surface as obtained from segmentation of the ablation volume defined by contrast-enhanced CT.

**TABLE C1.** Results of Experiments without A Simulated Vessel

Exp num	Electrode Size (cm)	Power (W)	Duration (s)	Mean Error (mm)	Max Error (mm)
1	4	80	800	1	2.3
2	4	80	800	0.6	1.8
3	4	115	600	0.4	1.6
4	4	80	600	0.7	2.9
5	5	120	600	1.6	4.1
6	5	140	600	1.8	4.9
7	3	60	600	0.6	1.7
8	3	60	480	1.1	2.9
9	2	50	120	0.5	1.6
10	4	60	120	0.9	1.6

Electrode size is the diameter of deployed tines. Power is the RF power set on the generator Duration is the time that the power was applied. The average and maximum errors refer to the distance between the computed ablation volume surface and the actual ablation volume surface as obtained from segmentation of the lesion on the scanned agar slice.

**TABLE C2.** Results of Experiments with a Simulated Vessel

Exp Num	Electrode Size (cm)	Power (W)	Duration (s)	Vessel Offset (mm)	Mean Error (mm)	Max Error (mm)
11	5	120	600	20	0.8	2.8
12	5	120	600	25	1.7	3.6
13	5	120	600	30	1.1	4
14	5	120	600	35	0.9	2.1
15	4	90	480	20	0.6	2.2
16	5	120	600	25	0.8	3.4
17	5	120	600	30	0.6	1.8

Vessel offset is the distance of the simulated vessel from the ablation probe; average and maximum errors refer to the distance between the computed ablation volume surface and the actual ablation volume surface as obtained from segmentation of the lesion on the scanned agar slice.

**TABLE C3.** Results from the In Vivo Porcine Model

Ablation	Electrode Size (cm)	Maximum Error (mm)	
		Computer Model	Manufacturer’s Model
1	3	3.6	7.1
2	3	5.0	10.0
3	3	5.1	6.6
4	3	4.3	7.5
5*	3	8.1	9.3
6	3	5.0	6.3

* Measurements were complicated by the ablation crossing a fissure.

## Abbreviations:

HCC: hepatocellular carcinoma
RFA: radiofrequency ablation
CT: Computed Tomography
GPU: Graphics Processing Unit
